# Indolent primary effusion lymphoma

**DOI:** 10.1002/jha2.43

**Published:** 2020-07-21

**Authors:** Vidya Nagrale, Ahmad Alduaij, Ahmad Alrustamani, Lois Richard

**Affiliations:** ^1^ Sheikh Khalifa Medical City Abu Dhabi UAE; ^2^ Cleveland Clinic Abu Dhabi Abu Dhabi UAE

A 79‐year‐old woman presented with shortness of breath and paroxysmal nocturnal dyspnoea since 4 months. The patient has a history of hypertension, heart failure, and a renal transplant 16 years ago for hypertensive nephrosclerosis maintained with mycophenolate mofetil and ciclosporine. Physical examination and chest radiograph showed an extensive right pleural effusion. Therapeutic thoracentesis was performed. Pleural fluid cytology showed large pleomorphic cells with high nuclear‐cytoplasmic ratio, fine granular chromatin, irregular nuclear membrane, multiple nucleoli, and scant deep basophilic occasionally vacuolated cytoplasm (Fig. 1). Immunohistochemical stains were positive for CD38, CD30, CD43, CD45 (variable), epithelial membrane antigen, and human herpes virus 8 (HHV8)^+^ (Fig. 1); and negative for CD138, CD20, Pax‐5, and CD3, and Epstein‐Barr virus (EBV) by chromogenic‐in situ‐hybridization. Serologic studies for human immunodeficiency virus (HIV) and hepatitis C virus were negative, EBV showed a past infection with viral capsid antibody‐immunoglobulin [M] VCA^‐^IgM^‐^, VCA^‐^IgG^+^, and Epstein‐Barr Nuclear Antigen (EBNA) antibody^+^. EBV Real‐time polymerase chain reaction viral load study was negative. A diagnosis of post‐transplant primary effusion lymphoma (PEL) in an HIV negative patient was rendered. A concurrent bone marrow was not involved. The patient was lost to follow‐up and presented 17 months later with shortness of breath and a large ascites without pleural effusion. There were no B symptoms. Peritoneal fluid cytology findings were similar to the diagnostic pleural fluid. Flow cytometry showed neoplastic cells with the following immunophenotype: CD45^+^, CD38^+^, HLA‐DR^+^, myeloid^‐^, T^‐^, and B^‐^. A whole‐body computed tomography showed no tumor masses. Patient refused chemotherapy, improved on drainage/minimal immunosuppression, continues follow‐up with no new complaints, and is alive and well 30 months from initial diagnosis without antineoplastic treatment.

**FIGURE 1:**
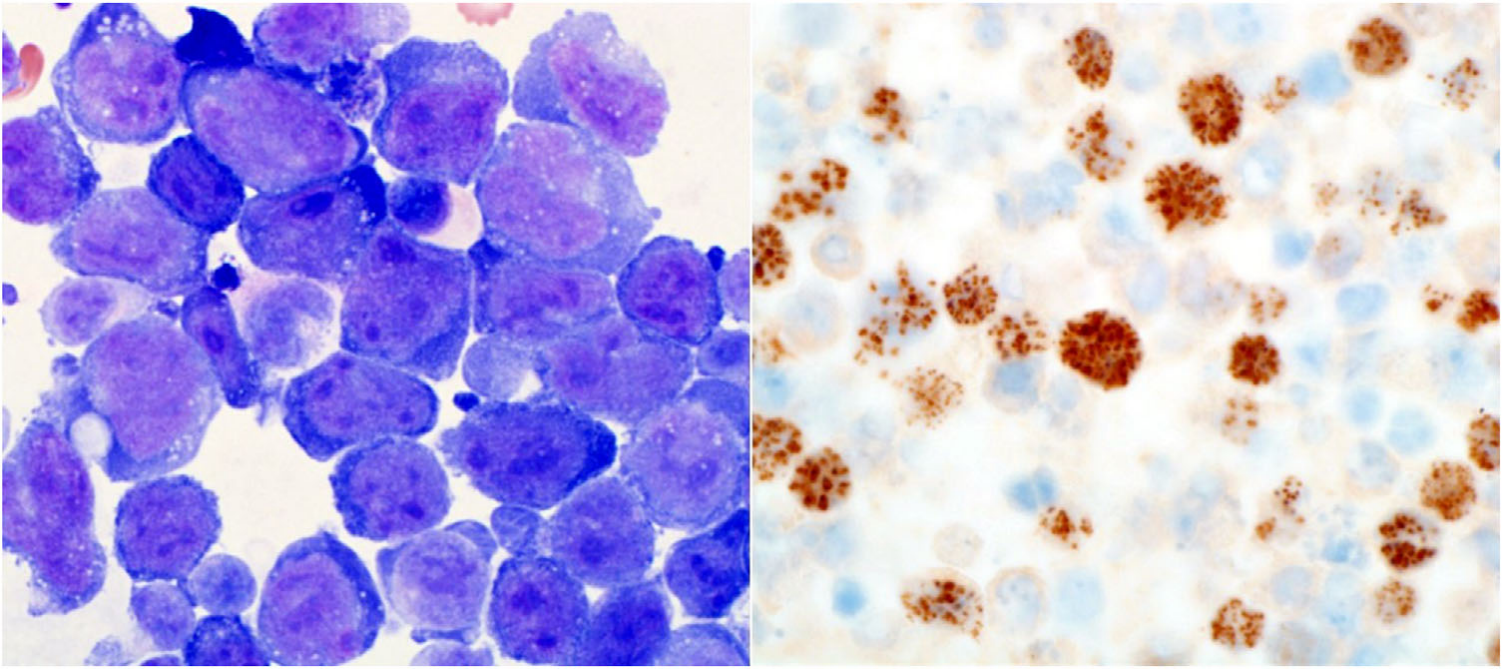
Pleural fluid cytology. On the left, May–Grünwald–Giemsa stained pleural fluid cytospin smear showing pleomorphic lymphoma cells (original magnification ×1000 oil immersion). On the right, cell block shows lymphoma cells positive for immunohistochemical stain human herpes virus 8 (original magnification ×400 oil immersion).

This case is a rare example of an indolent post‐transplant metachronous pleural and peritoneal primary effusion lymphoma. The clinical behavior illustrates how HHV8‐driven neoplasms including primary effusion lymphoma may be more heterogeneous than presently classified especially in post‐transplant HIV‐negative elderly patients. More studies are necessary for clinical and therapeutic stratification of these cases.

## AUTHOR CONTRIBUTIONS

Vidya Nagrale designed, performed research, drafted and finalized the manuscript. Ahmad Alduaij provided clinical and pathology input from Cleveland Clinic Abu Dhabi. Ahmad Alrustamani provided clinical follow‐up and reviewed the manuscript. Lois Richard photographed images and reviewed the manuscript.

## CONFLICT OF INTEREST

The authors declare no conflict of interest.

